# Supplementary material for: Exploring the hydrophobic effects of quaternary ammonium copolymers on corrosion of casing and tubing steel in acidic solution
Source: PLoS One. 2025 Jun 18;20(6):e0320981. doi: 10.1371/journal.pone.0320981 (PMC12176120; doi:10.1371/journal.pone.0320981)
Supplement: S1 File — Figure SI-1. FTIR spectrum of quaternary ammonium bromide 3. Figure SI-2. (a) 1H and (b) 13C NMR spectra of quaternary ammonium bromide 3 in D2O. Figure SI-3. FTIR spectrum of homopolymer 5. Figure SI-4. 1H NMR spectra of (a) monomer 4 and (b) homopolymer 5 in D2O. Figure SI-5. 13C NMR spectra of (a) monomer 4 and (b) homopolymer 5 in D2O. Figure SI-6. FTIR spectrum of copolymer 6b. Figure SI-7. FTIR spectrum of copolymer 6b. Figure SI-8. 1H NMR spectra of (a) monomer 4 in D2O, and (b) 6b in CD3OD. Figure SI-9. 13C NMR spectra of (a) monomer 4 in D2O, and (b) 6b in CD3OD. Figure SI-10. TGA curves of homopolymer 5 and copolymers 6a and 6b. Figure SI-11: SEM images (at 500x) of corroded P110 CS in 15% HCl for 6h (a) in the absence and presence of (b) 5 (c) 6a and (d) 6b. Figure SI-12: SEM images (at 1000x) of corroded P110 CS in 15% HCl for 6h (a) in the absence and presence of (b) 5, (c) 6a and (d) 6b. Figure SI-13: SEM images (at 2000x) of corroded P110 CS in 15% HCl for 6h (a) in the absence and presence of (b) 5, (c) 6a and (d) 6b. Figure SI-14: Pictures showing sites taken for EDX analyses from corroded P110 CS samples (a) in the absence and presence of (b) 5, (c) 6a and (d) 6b. (DOCX) [file pone.0320981.s001.docx]

**SUPPORTING INFORMATION**

**Exploring the Hydrophobic Effects of Quaternary Ammonium Copolymers on Corrosion of Casing and Tubing Steel in Acidic Solution**

Ghadeer Mubarak^1^, Chandrabhan Verma*^1^, Mohammad A. Jafar Mazumder^2,3^, Imad Barsoum^4^, Akram Alfantazi^1^**

^1^Department of Petroleum and Chemical Engineering, Khalifa University of Science and Technology, P.O. Box 2533, Abu Dhabi, United Arab Emirates

^2^ Department of Chemistry, King Fahd University of Petroleum & Minerals, Dhahran 31261, Saudia Arabia

^3^ Interdisciplinary Research Center for Refining and Advanced Chemicals, King Fahd University of Petroleum & Minerals, Dhahran 31261, Saudi Arabia.

^4^ Department of Mechanical Engineering, Khalifa University of Science and Technology, P.O. Box 2533, Abu Dhabi, United Arab Emirates

**Corresponding Author’s**:

E-mail:

[chandraverma.rs.apc@itbhu.ac.in](mailto:chandraverma.rs.apc@itbhu.ac.in) (CV);

[akram.alfantazi@ku.ac.ae](mailto:akram.alfantazi@ku.ac.ae) (AA);

**Figure SI1.** FTIR spectrum of quaternary ammonium bromide quaternary ammonium bromide (3).

**Figure SI2.** (a) ^1^H and (b) ^13^C NMR spectra of quaternary ammonium bromide quaternary ammonium bromide (3) in D_2_O.

**Figure SI3.** FTIR spectrum of homopolymer **5**.

**Figure SI4.** ^1^H NMR spectra of (a) monomer **4** and (b) homopolymer **5** in D_2_O.

**Figure SI5.** ^13^C NMR spectra of (a) monomer **4** and (b) homopolymer **5** in D_2_O.

**Figure SI6.** FTIR spectrum of copolymer **6b**.

**Figure SI7.** FTIR spectrum of copolymer **6b**.

**Figure SI8.** ^1^H NMR spectra of (a) monomer **4** in D_2_O, and (b) **6b** in CD_3_OD.

**Figure SI9.** ^13^C NMR spectra of (a) monomer **4** in D_2_O, and (b) **6b** in CD_3_OD.

**Figure SI10.** TGA curves of homopolymer **5** and copolymers **6a** and **6b**.

**Figure SI-11**: SEM images (at 500x) of corroded P110 CS in 15% HCl for 6h (a) in the absence and presence of (b) **5** (c) **6a** and (d) **6b**.

**Figure SI-12**: SEM images (at 1000x) of corroded P110 CS in 15% HCl for 6h (a) in the absence and presence of (b) **5**, (c) **6a** and (d) **6b**.

**Figure SI-13**: SEM images (at 2000x) of corroded P110 CS in 15% HCl for 6h (a) in the absence and presence of (b) **5**, (c) **6a** and (d) **6b**.

**Figure SI-14**: Pictures showing sites taken for EDX analyses from corroded P110 CS samples (a) in the absence and presence of (b) **5**, (c) **6a** and (d) **6b**.
